# Study protocol of coaching end-of-life palliative care for advanced heart failure patients and their family caregivers in rural appalachia: a randomized controlled trial

**DOI:** 10.1186/s12904-019-0500-z

**Published:** 2019-12-29

**Authors:** Ubolrat Piamjariyakul, Trisha Petitte, Angel Smothers, Sijin Wen, Elizabeth Morrissey, Stephanie Young, George Sokos, Alvin H. Moss, Carol E. Smith

**Affiliations:** 1 West Virginia University, School of Nursing Health Sciences Center, Post Office Box 9600 – Office 6701, Morgantown, WV 26506-9602 USA; 2 Department of Biostatistics School of Public Health, West Virginia University, Morgantown, USA; 3 Advanced Heart Failure, West Virginia University Heart and Vascular Institute, J.W. Ruby Memorial Hospital, Morgantown, USA; 4 Sections of Nephrology and Supportive Care, West Virginia University Center for Health Ethics and Law, Morgantown, USA; 5 University of Kansas Medical Center, School of Nursing and School of Preventive Medicine, Morgantown, USA

**Keywords:** Heart failure, End-of-life, Palliative care, Study protocol, Randomized controlled trial, Appalachia

## Abstract

**Background:**

Heart failure (HF) afflicts 6.5 million Americans with devastating consequences to patients and their family caregivers. Families are rarely prepared for worsening HF and are not informed about end-of-life and palliative care (EOLPC) conservative comfort options especially during the end stage. West Virginia (WV) has the highest rate of HF deaths in the U.S. where 14% of the population over 65 years have HF. Thus, there is a need to investigate a new family EOLPC intervention (FamPALcare), where nurses coach family-managed advanced HF care at home.

**Methods:**

This study uses a randomized controlled trial (RCT) design stratified by gender to determine any differences in the FamPALcare HF patients and their family caregiver outcomes versus standard care group outcomes (*N* = 72). Aim 1 is to test the FamPALcare nursing care intervention with patients and family members managing home supportive EOLPC for advanced HF. Aim 2 is to assess implementation of the FamPALcare intervention and research procedures for subsequent clinical trials. Intervention group will receive routine standard care, plus 5-weekly FamPALcare intervention delivered by community-based nurses. The intervention sessions involve coaching patients and family caregivers in advanced HF home care and supporting EOLPC discussions based on patients’ preferences. Data are collected at baseline, 3, and 6 months. Recruitment is from sites affiliated with a large regional hospital in WV and community centers across the state.

**Discussion:**

The outcomes of this clinical trial will result in new knowledge on coaching techniques for EOLPC and approaches to palliative and end-of-life rural home care. The HF population in WV will benefit from a reduction in suffering from the most common advanced HF symptoms, selecting their preferred EOLPC care options, determining their advance directives, and increasing skills and resources for advanced HF home care. The study will provide a long-term collaboration with rural community leaders, and collection of data on the implementation and research procedures for a subsequent large multi-site clinical trial of the FamPALcare intervention. Multidisciplinary students have opportunity to engage in the research process.

**Trial registration:**

ClinicalTrials.gov NCT04153890, Registered on 4 November 2019

## Background

Heart failure (HF) afflicts 6.5 million Americans [1] with devastating consequences to patients and their family caregivers, [2] especially during severe symptoms in the end stage. Advanced HF was defined by American Heart Association (AHA) as “the presence of progressive and/or persistent severe signs and symptoms of HF despite optimized medical, surgical, and device therapy [3].” When patients and family members are not prepared for worsening HF and are not informed about end-of-life and palliative care (EOLPC) conservative comfort options, they experience depression, fear of painful death, home care burden, and medical expenses from anxiously seeking aggressive but futile care [4]. Notably, West Virginia (WV) has the highest rate of HF deaths in the U.S. at 32.6 per 100,000 population, [5] where 14% of those are over 65 years have HF. WV is in the Appalachian mountainous region, a high priority for research as residents experience extreme health and poverty inequities and limited access to healthcare [6]. Furthermore, home EOLPC is lacking across this disadvantaged rural area [7]. Thus, there is a need to investigate a new family intervention (FamPALcare), where the nurses coach family-managed advanced HF care at home.

Palliative care is a team-approach, patient-centered comprehensive treatment of the discomfort, symptoms, and stress of serious illness with the goals of preventing and relieving suffering, and of improving quality of life for patients and their families through early identification, correct assessment, and treatment of pain and other problems, whether physical, psychological, and/or spiritual [8, 9]. WVU Center for Palliative Care adds that palliative care should be provided regardless of patient’s stage of disease or the need for other therapies, in accordance with patient’s values and preferences [10].

This study addresses the National Institutes of Health Academic Research Enhancement Award (AREA) priorities for conducting a low risk clinical trial to provide a foundation to advance scientific EOLPC knowledge and testing of our intervention efficacy in a larger clinical trial. Additionally, effective EOLPC interventions are priorities of palliative care professionals and palliative care needs must be addressed with vulnerable and advanced HF patients and their families [11, 12]. This study also addresses the priority problem of the lack information for families providing advanced HF home care and preventing unwanted and unwarranted rehospitalizations at the advanced stage of HF.

In FamPALcare intervention being used in this study, nurses coach by information sharing and guiding patient and family members to gain knowledge to manage the frequent distressful advanced HF decline symptoms (breathlessness, anxiety, depression, fatigue). Innovation of the study includes gaining data about the knowledge, and limited resources available for rural EOL care. The FamPALcare materials were designed for low literacy, which is a major barrier to serious illness care and is associated with poor health outcomes [13].

An important innovation of this intervention is the component addressing family caregivers’ needs. A systematic review [14] showed a growing number of studies verifying caregiver contributions to HF home care. However, all these studies are at an early stage of scientific research. Thus, the FamPALcare intervention provides family caregivers with the knowledge and practical skills to monitor both the patient’s advancing HF status and their own depression and home care burden [15, 16]. Through coaching, family caregivers can develop greater confidence in providing HF home EOLPC and ensure that the intervention reflects the patients’ and families’ preferences [17].

Overall, previous testing in these studies demonstrated that a coaching approach was feasible, [15] provided HF patients and caregivers with practical skills to partner with professionals on EOLPC, increased home management of patients’ breathlessness symptoms, [18] and reduced family caregiver burden and out-of-pocket costs due to fewer rehospitalizations [19]. In this study, all previously tested components are combined into our novel FamPALcare intervention with significant approaches for rural families residing in Appalachia.

### Conceptual model

Coaching Model for End-of-Life and Palliative Care Research was used to guide selection of coaching components, procedures, and measures targeted to accomplish the study aims [20, 21]. This model depicts relationships among clinical, psychological, and economic factors affecting home EOLPC care. This model guides nurses to coach family discussions of advance directives and conservative management of HF home EOLPC options. It measures HF status and family caregivers’ burden and provides resources needed for HF care.

## Methods/design

### Aims

The overall objective is to test whether the 5-weekly coaching FamPALcare intervention educational and supportive sessions will improve rural home EOLPC for advanced HF at the 6 month follow up. Specific aims, hypotheses, and objectives are listed in Table 1.

**Table 1:** List of Specific Aims, Hypotheses, and Objectives for the Study

Specific Aim 1: Test the FamPALcare nursing care intervention with patients and family members managing home supportive EOLPC for advanced HF in rural WV. *Hypothesis 1a.* The intervention group HF patients will rate greater improvement on: (a) confidence and preparedness in management of their severe HF (status, breathlessness, anxiety, and depression); (b) increased numbers of signed decisions about their preferred EOLPC options for advanced HF; (c) increased numbers of signed advance directives, compared to control group patients at 6 months. *Hypothesis 1b.* The intervention group family caregivers will rate greater improvement on: (a) quality of life (physical, mental), (b) confidence and preparedness in providing home HF EOLPC, and (c) home care burden, compared to control group caregivers at 6 months. Specific Aim 2: Assess implementation of the FamPALcare intervention and research procedures for subsequent clinical trials. *Aim 2a.* Evaluate the quality of EOLPC of the FamPALcare intervention measured via (1) patient/family caregiver intervention helpfulness questionnaire responses; (2) multidisciplinary healthcare professionals’ and rural community leaders’ focus group research on the FamPALcare outcomes; and (3) determination of FamPALcare implementation cost using traditional tabulated cost minimization analysis. *Aim 2b.* Collect participant recruitment, enrollment, and retention rates to determine future recruitment strategies and understand reasons for non-participation to maintain good rates in rural clinical studies. The fidelity of the FamPALcare intervention will be assessed using trained research nurse observer checklists.	

### Design/methodology

This study uses a randomized controlled trial (RCT) design stratified by gender [22] to determine any differences in the FamPALcare HF patients and their family caregiver outcomes versus standard care group outcomes (*N* = 72) (Table 2).

**Table 2:** FamPALcare Coaching Intervention (X_1_-X_7_) Sequence with Data Collection (O_1_-O_3_)

Random Group Assignment	FamPALcare Intervention and Standard Care Groups		Follow-up Data Collection Post-Intervention	
	Baseline Data	FamPALcare Intervention	3 months Booster	6 months
Group 1 FamPALcare Intervention	O_1_	X_1_–X_5_ Weekly intervention across 5 weeks	X_6_, O_2_ Reinforcement on EOLPC options	X_7_, O_3_ Evaluation of FamPALcare
Group 2 Standard Care	O_1_	Standard care	O_2_	O_3_

Note. O_1_-O_3_ = observation, data collection time points; X_1_–X_5_ = FamPALcare coaching intervention of weekly home visits. Nurse-administered and conducted across 5 weeks, X_6_ = Booster Reinforcement at 3 months and reinforcement on each family selected conservative EOLPC options for patients and family members. X_7_ = Evaluation of FamPALcare

#### Randomization

Our statistician consultant will use computer-generated random numbers for subject enrollment. To ensure a balance design with gender, two equal randomization lists will be used one for male and one for female. Within each gender group the families will be randomly assigned into either control or intervention groups in a 1:1 fashion. The group assignment will be placed in the sealed envelope under each gender group. Our RCT design is consistent with the CONSORT standards, [23] and rigorous research procedures. Recruiters will be blinded to group assignment until informed consents are signed and after a group random allocation of each patient/caregiver dyad [24, 25]. The proposed design addresses threats to both internal and external validity and is robust for detecting differences between small groups [26].

#### Characteristics of participants

The sample includes adult (≥18 years) advanced HF patients (NYHA III and IV and Stages C and D) [3] and their family caregivers who are involved in daily home care will be recruited. Both patients and caregivers will provide consent, and will be randomly assigned to a study group as a dyad.

#### Eligibility criteria

Inclusion criteria are dyads of family caregivers and patients with advanced systolic and diastolic HF. All participants must be alert and oriented, provide written consent, and be able to read and write in English. Family caregivers are those designated by the HF patient as non-paid primary persons who assists with HF home care, thus not requiring the dyad to be spouses. Exclusion criteria are patients who already received or are on a waiting list for a heart transplant or left ventricular assist device (LVAD); and those with other terminal illness or dementia, such as Alzheimer’s disease. Also excluded are those caregivers with a disability that precludes their use of FamPALcare intervention materials such as those suffering with Alzheimer’s disease.

#### Sample size

There will be 18 patient-caregiver dyads per each group (total *N* = 72; 36 patients and 36 caregivers). Sample size is based on calculated breathlessness, [18] the main patient outcome measured, and the HF patients’ and their family caregivers’ greatest concern [27, 28]. This sample size will have at least 80% power to detect a reduction of one SD using one-sided two-sample t-test at a significance level of 0.05. Further, a cardiologist, pulmonologist, and two palliative physicians experienced in caring for patients with EOL breathlessness agreed that even a 0.5 standard deviation (SD) improvement is an indication of best possible management of advanced HF breathlessness [29]. This sample size is also accounted for an expected 20% attrition (due to HF deaths).

#### Settings, recruitment and retention plans

Successful strategies for recruiting research participants in rural Appalachia will be used [30]. Recruitment is from sites affiliated with a large regional hospital in WV including Heart and Vascular Institute, local health centers or clinics, churches, physician offices and community centers throughout the state. The cardiology nurse study coordinator will identify potential participants prospectively through daily reviews of hospital and outpatient records for all HF patient hospitalizations and clinic visits. Personnel who already have clinic responsibilities and access to patient records, consistent with HIPAA requirements, will perform subject eligibility screening and the initial recruitment contact. Nurse researchers who are active in our rural communities, will engage these communities in recruitment. There will be an expected delay in enrollment during winter (December to March) due to extreme weather and road conditions in rural Appalachia. Our study coordinator will oversee participant timelines, monitor, and report the progress of enrollment on quarterly basis.

All procedures outlined in retention strategies were successful in prior longitudinal clinical trial studies [31]. Retention has been related to study recruitment being performed by persons with similar characteristics to those recruited. Having Appalachian community or faith-based nurses for intervention implementation will promote the engagement in the coaching sessions. To enhance the retention rate, these research nurses will develop trust and rapport and provide flexibility in the intervention scheduling.

### Interventions

#### Standard care group

The standard care group will receive routine HF instruction during hospital or clinic visits. All patients can be referred for palliative care consults with an individual from the WVU palliative care team if requested (all consults will be tabulated). All patients in this study will have standard HF care available through the WVU hospital and outpatient clinics, prescribed by the patient’s cardiologist. Standard HF care includes materials routinely given to all patients with HF at the hospital or outpatient clinics.

#### FamPALcare intervention group

The FamPALcare intervention group will receive standard care, plus FamPALcare intervention delivered by community-based nurses. FamPALcare intervention involves coaching patients and family caregivers in advanced HF home care and supporting EOLPC discussions. FamPALcare intervention will begin by using recent American Heart Association (AHA) study recommendations and the “Conversation Ready” for wording when approaching the topic of EOL care [32] as a guide along with an illustrated advanced HF trajectory graph [20]. The nurse will coach the patient and family in making decisions about EOLPC options based on their preferences. Nurses will go over advance directive forms and recommend that forms be signed and taken to their next doctor appointment. Intervention participants will receive five coaching sessions with telephone follow-up to reinforce HF home care.

Our intervention nurses reinforce following the prescribed medication and diet, timely symptom reporting, family caregivers accepting help from others, sharing emotions with trusted others, and taking short naps to support their own health [33]. Referrals to local support programs (i.e. church minister, volunteers, or faith-based nurses) are made with the dyad’s permission. Applications for low-cost prescription drug programs will be provided upon request. The number of referrals who were contacted will be tracked to identify local resources used. Because involvement of multiple family caregivers is a common strength among Appalachians, secondary family caregivers involved in home HF care will be allowed to attend the intervention session. We will keep track of how many have secondary family caregivers participate.

During the sessions, the nurse will use the “teach-back” process (“*please describe what you learned today*”) to verify understanding at the end of each FamPALcare discussion [15]. Then the nurse interventionist will tabulate and report on content needing reinforcement. A follow up telephone calls will be conducted at 3 months to reinforce the practice of FamPALcare.

#### Treatment fidelity and quality assurance procedures

Fidelity will be monitored by the PI using a fidelity rating scale. Fidelity rating is performed by observation during the intervention to ensure the reliable and valid implementation of the FamPALcare intervention [34]. For quality assurance measures, training sessions on appropriate communication techniques and following research protocols will be provided to the nurse interventionists and data collectors.

### Data collection

Data will be collected from all patients and caregivers at baseline, 3 months, and 6 months. The nurse will obtain data from patients and caregivers separately, emphasizing privacy and importance of each subjects’ independent answers [24]. None of the intervention or data collection nurses will be the HF patients’ usual healthcare providers. To preclude diffusion of treatment across groups, the research nurses working with the intervention group will be different from nurses with the control group.

#### Data management and quality assurance

Quality assurance and data integrity techniques include data management protocols and an audit trail of the data management decisions. Other data integrity techniques include developing guides for verification of data, coding each subject’s data, analyzing for data distributional features of group equivalencies at baseline and meeting statistical assumptions prior to quantitative analyses, and the need for transformations [35]. Differences in age, gender, or demographics between participants and those who do not choose to participate will be reported. Reasons for refusal to participate are recorded. Rules for managing missing data will be discussed with our biostatistician consultants to examine missing at random or repeated missing data and will be reported in publications. Missing data will be identified and recollected within 2 weeks upon data check-in. Research staff will use a *REDCap* survey to complete all data entry. Parameters to identify ranges and options for “decline to answer” will be used to prevent and monitor invalid or missing data. Data conversion from *REDCap* to SPSS/SAS will be conducted on a weekly basis. The CONSORT enrollment diagram will be updated quarterly and reported in our research team and Safety Monitoring Committee (SMC) meetings.

### Statistical analyses

The consulting biostatistician will guide the use of intent-to-treat statistical approaches for all patient and caregiver outcomes. All participants will complete demographic data at baseline. All instrument measures have established discriminant or construct validity, internal consistency, reliability, and specificity. Each has been used with diverse populations, chronic illness patients, and with HF families. The measures distinguish clinically significant differences and are sensitive to change over time [36]. As pretested, these questionnaires were easily completed within 15–20 min.

#### Data analysis for specific aim 1: hypothesis 1a & 1b

Table 3 illustrated the measures and instrument for addressing Specific Aim 1, hypotheses 1a and 1 b. Hierarchical linear models will be used to compare groups in the presence of repeated measures and nested family members. Models will include the fixed effects of treatment, time, and the treatment-time interaction, with patient and family caregiver effects treated as random to account for the dependence among repeated observations on the same subject and among observations on subjects within the same family, respectively. Models will identify any changes in the outcome variables over time, for differences in outcomes between the groups, and for any within-group differences over time [46]. The SAS Proc MIXED procedure will be used. General linear hypothesis tests will be used to test for significant differences using linear contrasts of selected model parameters. Point estimates and confidence intervals will also be generated using contrasts and estimates of the appropriate linear combinations of model parameters. For multiple testing, the overall experimental error rate will be conserved by correcting the per-test error rate using the Bonferroni procedure. Caregivers’ daily involvement, as measured by burden scale, will be used as a potential covariate. The numbers of mental health referral (for severe anxiety and depression) will be tracked to compare between groups.

**Table 3:** Measures and Instrument for Specific Aim 1 (Data collection at baseline, 3, and 6 months)

Measures: Specific Aim 1, Hypothesis 1a, 1b	Operationally Defined
1a. Patient’s Kansas City Cardiomyopathy Questionnaire (KCCQ) [37] 12-item Likert.^a, c, d, e^ [completed by patient]	Management of HF status, HF-related symptoms (i.e. breathlessness) and physical function status, α = 0.90.^a^
1a. Tabulate proportion of patients selecting HF EOLPC options and signing advance directives, and.	Identify preferred HF conservative care options. Signed directives and confirmed EOLPC options
1a.1b Patient Health Questionnaire (PHQ-4) Scale, [38] 4-item Likert.^a, b,e^ [completed by patient & caregiver]	Assess patient’s/family caregiver’s depression and anxiety, α = 0.82.^a^ Referral will be made [39].
1a.1b HF Home-Care Skills, [40] 9-item Likert.^a, b, d, e^ [completed by patient & caregiver]	HF home care skills (i.e., if the patient’s legs/ feet are swollen, I contact MD/nurse), α = 0.80.
1a.1b. Confidence in HF home care, [41] 4-item multiple choice.^a, e, f^ [completed by patient & caregiver]	Perceived confidence in providing home HF EOLPC, α = 0.87^a^ *“How confident are you in managing your worsening HF at home?”*
1a.1b. Preparedness for HF EOLPC Home Care, [42] 1-item Likert.^c, e^ [completed by patient & caregiver]	Perceived readiness/ability to manage home HF EOLPC.
1b. Caregivers’ quality of life (QoL) SF12v2, [43] 12-item Likert ^a, c, d, e^ [completed by caregiver]	Caregivers’ physical and mental health outcomes. α = 0.90 to 0.93.
1b. Short-form Zarit Caregiver Burden Interviews, [44, 45] 12-item Likert.^a, c, d, e^ [completed by caregiver]	Record physical, social, financial, and emotional components of home caregiving burden, α = 0.89.^a, b^

Questionnaire Reliability^a, b, c^ & Validity^d, e, f^; ^a^Cronbach’s alpha with HF patients; ^b^Cronbach’s alpha with healthy population; ^c^Reliability reported for adults with chronic illnesses >0.70; ^d^Factor analysis loadings of subscales >0.35; ^e^Established concurrent validity using correlation with other instruments or clinical ratings in known groups; ^f^Published norms or ranges

To determine differences between groups on HF EOLPC decisions, the number of family members and patients who sign an advance directive form, make a decision, or identify someone who helps them decide on HF EOLPC options will be tabulated and compared. The proportion of patients signing an advance directive form will be used to evaluate the acceptability of EOLPC discussions. A nurse blinded to group, will adjudicate the patients’ hospitalizations, ER visits, and causes of death within the time period to determine if the hospitalizations and deaths are due to HF. Initial analysis using a chi-square test (or Fisher’s exact, if event rates are small) will compare groups on the proportion of subjects who die or who are rehospitalized due to HF. Kaplan-Meier method will be used to estimate survival curves (i.e., probability of remaining event-free) up to the last follow-up [47]. Cox proportional hazards regression model will be used to assess the survival for group differences [48]. Subjects’ outcomes will be recorded at the time of death or last follow-up such as 6 months post-intervention, whichever comes first.

#### Data analysis for specific aim 2 (2a & 2b)

##### Specific aim 2a

Aim 2a is to evaluate the quality of EOLPC of the FamPALcare intervention measured via (2a.1) patient/family caregiver intervention helpfulness questionnaire responses; (2a.2) multidisciplinary healthcare professionals’ and rural community leaders’ focus group research on the FamPALcare outcomes; and (2a.3) determination of FamPALcare implementation cost using traditional tabulated cost minimization analysis [49].

For Aim 2a.1*,* each patient and family member will rate the helpfulness of each intervention component on an 11-item Likert-type scale [15] at 3 months following the FamPALcare intervention. Response options range from: not helpful (1) to very helpful (5). Sample questions are: (a) Do you believe the nurse discussions helped with your HF home care skills? (b) Do you think the nurse discussions helped you to manage breathlessness and prevent rehospitalizations? (c) Did discussion about EOLPC options help your family decide on a EOLPC plan? A descriptive analysis will be used to analyze the family members’ survey responses on the helpfulness scale.

For Aim 2a.2*,* multidisciplinary team members and rural community leaders will complete the 8-item FamPALcare intervention helpfulness scale. Sample helpfulness questions are: (a) Do you believe that patients and their family members received helpful information to prepare them for their advanced HF? (b) Do you believe that the FamPALcare intervention contains culturally sensitive content and examples? Response options range from: strongly disagree (1) to strongly agree (5). A descriptive analysis will be used to analyze the responses on the helpfulness scale. The team will discuss topics where families required reinforcement that arose during the intervention including the teach-back sessions, to improve the intervention. These topics will be tabulated and reported.

Also, multidisciplinary healthcare professional team members and rural community leaders will be invited to participate in focus groups to evaluate the EOL intervention and their likelihood to initiate EOLPC coaching approaches in their own practice. The focus group will be held in the West Virginia University School of Nursing conference room, equipped with a telecommunication system enabling our community team members from rural areas to participate. The PI will conduct the focus group discussion with probing questions for clarification and depth of opinions and the Co-I will facilitate the focus group discussion and take notes. The focus group discussion will be audiotaped and data transcriptions will be summarized with no individual data source disclosed. The focus groups of our previous studies were typically completed in 90 min when data saturation was achieved (no new topics or information is brought up). The lead facilitator has experience in focus group research methods and working with HF patients and caregivers.

Content analysis research methods will be used to categorize the participants’ focus-group data transcriptions. Content analysis is a method used to identify the meaning and relationships of words or concepts of spoken words transcribed into written narrative data [50]. To ensure the consistency of data interpretation, two researchers will independently categorize the data and then meet to compare content and resolve any differences in topic categorization. Data will be summarized in repeating themes and topics using participants’ own words, which demonstrates integrity and authentic data [51].

For Aim 2a.3*,* to determine costs of the FamPALcare intervention implementation, all charges related to implementation will be calculated. This includes costs of personnel time for administering each discussion session, the materials, home/clinic visit travel, and telephone and mailing costs. The research costs of data collection and survey photocopying will be totaled and reported as study costs. The average intervention implementation cost across all families will be reported. In terms of potential family out-of-pocket cost savings, the number of HF related hospitalizations and emergency room (ER) visits will be tallied per patient. The cost of all HF-related hospitalizations and ER visits 6 months prior to the intervention will be compared to 6-month post-intervention using the average cost of a 20% co-pay for a 4-day hospital stay (the average HF hospital length of stay) [19] per admission. Patient’s medical record review (per IRB approval) to determine if the hospitalizations/ER/deaths are HF related or not will be adjudicated by the nurse blinded to group assignment.

#### Specific aim 2b

Specific Aim 2 b is to collect participant recruitment, enrollment, and retention rates to determine future recruitment strategies and understand reasons for non-participation to maintain good rates in rural clinical studies. Descriptive statistics with bar-plots and boxplots will be used for analysis. Participant recruitment, enrollment, and retention rates will be tabulated and reported. We will use nurse interventionist observation checklists. The fidelity of the FamPALcare intervention will be assessed using trained research nurse observer checklists. Fidelity will be rated and addressed to ensure all EOLPC options are discussed.

### Human subject protection and Management of Risks to subjects

The research team at West Virginia University (WVU) main campus and at WVU affiliated hospitals and clinics will conduct subject recruitment. All research staff who are involved in subject recruitment, medical records reviews, and data analyses will be trained and will complete the NIH-approved Human Subjects Protection Certification through the Collaborative Institutional Training Initiative (CITI Program). All study participants (family members and patients) will consent to the study prior to participating. A HIPAA waiver will be obtained prior to obtaining a recruitment list from the hospital. All the research staff will abide by all tenets of WVU’s confidentiality policies, including privacy-protection standards for research subjects.

Several procedures are established for protecting against the risk of breaking confidentiality. Information-protection procedures specific to data management, communications, and the electronic environment are also in place. The questionnaires will be kept in a locked file cabinet in the research office at all times. Consent forms are filed separately from the data. Electronic research-related data will be stored in the designated research network drive under at the university firewall-protected server, which is backed-up daily.

### Data and safety monitoring

All research staff will notify the PI within 24 h if any potential study safety risks arise. The PI then notifies the IRB within 24 h and notifies National Institute of Nursing Research (NINR) within 7 days of the investigator becoming aware of the event. The adverse events will be systematically monitored and reported. The Safety Monitoring Committee (SMC) is the monitoring entity for this RCT. The SMC includes two experts who are independent of the study protocol. The SMC will review each potential adverse event (AE) upon its occurrence (real-time) and bi-annually thereafter. The SMC will provide direction to conduct interim analyses, stopping guidelines, and address any ethical issues that may arise related to palliative care or end-of-life discussions, study safety and research risks. These recommendations and actions taken will be reported to the IRB and NINR.

### Pitfalls, limitations, and alternatives

Population in West Virginia is predominantly 93% Caucasian; however, we will make an effort to enroll other underrepresented minorities into the study. Our community nurses will continue to engage under-represented and under-served community leaders and professionals and provide guidance on identifying and inviting minority participants. Recruitment from community health centers will increase the generalizability of the findings to other Appalachian regions. Although HF has a high mortality rate, the previous trials with advanced HF involving family caregivers showed low attrition rate. Most attrition was due to patient death, with very few caregivers’ withdrawing after patient death out of appreciation of the continual support from study nurses for these families [16, 52]. Participation in this study is likely to increase as program brochures are available in local churches through lay ministers, faith community nurses, and in community center announcements. As in previous studies, nurses, physicians, dieticians, and social workers who participate in HF patient hospital discharges or ER visits will refer families to the program. Our study retention protocols include mailed appointment reminders and telephone calls; and preferences for caregiver involvement with flexible times for coaching sessions.

## Discussion

The primary aim of this clinical trial is to compare the FamPALcare HF patients’ and their family caregivers’ outcomes to standard care control families’ outcomes. The secondary aim is to assess the implementation of FamPALcare intervention and the research procedures to be used in a subsequent large multi-site clinical trial testing the intervention efficacy. The long-term goal is to provide home-based EOLPC interventions, including our new FamPALcare intervention, as an option to address the challenge of providing rural EOL care in WV. The EOLPC options discussed with families are grounded from evidence-based national clinical guidelines for advanced HF and on professionals’ recommendations.

The outcomes of this clinical trial will result in new knowledge on coaching techniques for EOLPC and culturally sensitive approaches to palliative and end-of-life rural home care. The HF population in WV will benefit from a reduction in suffering from the most common severe advanced HF symptoms, selecting their preferred EOLPC care options, determining their advance directives, and increasing skills and resources for advanced HF home care. Clinical findings of this study will be used to improve the FamPALcare intervention and research protocol for subsequent clinical trials to improve patients’ and their family caregivers’ home HF end-of-life and palliative care outcomes. The study will provide a long-term collaboration with rural community leaders, and collection of data on the implementation and research procedures for a subsequent large clinical trial of the FamPALcare intervention. Further, this trial will engage community leaders in the program evaluation which will promote collaborations for conducting future translational research. Multidisciplinary students have opportunity to engage in the research process.

The PI and research team will comply with the NIH Policy on Dissemination of NIH-Funded Clinical Trial Information. The information about the clinical trial and the study results will be updated and made publicly available, in a timely manner, via ClinicalTrials.gov, a publicly accessible database operated by the NIH’s National Library of Medicine (NLM). Results from the trial will be submitted within 1 year after the trial’s primary completion date.

## Data Availability

The study plans to share data in aggregate and as overall study results.

## Abbreviations

AHA: American Heart Association
CONSORT: Consolidated Standards of Reporting Trials
EOLPC: End-of-life palliative care
FamPALCare: Family palliative care intervention
HF: Heart failure
IRB: Institutional Review Board
SMC: Safety Monitoring Committee
SPIRIT: Standard Protocol Items: Recommendations for Interventional Trials
WV: West Virginia
